# Suspected Brazilian Purpuric Fever, Brazilian Amazon Region

**DOI:** 10.3201/eid1504.090014

**Published:** 2009-04

**Authors:** Eucilene A. Santana-Porto, Adriana A. Oliveira, Marcos R.M. da Costa, Amiraldo Pinheiro, Consuelo Oliveira, Maria L. Lopes, Luiz E. Pereira, Claudio Sacchi, Wildo N. Aráujo, Jeremy Sobel

**Affiliations:** Ministry of Health, Brasília, Brazil (E.A. Santana-Porto, A.A. Oliveira, W.N. Aráujo, J. Sobel); Municipal Secretariat of Health, Anajás, Brazil (M.R.M. da Costa); Secretariat of Health of Pará State, Belém, Brazil (A. Pinheiro); Ministry of Health, Belém (C. Oliveira, M.L. Lopes); Instituto Adolfo Lutz, Secretariat of Health of São Paulo State, São Paulo, Brazil (L.E. Pereira, C. Sacchi); Centers for Disease Control and Prevention, Atlanta, GA, USA (J. Sobel)

**Keywords:** Amazon region, Brazil, children, hemorrhagic disease, purpuric fever, letter

**To the Editor:** Brazilian purpuric fever (BPF), a *Haemophilus aegyptius*–caused febrile hemorrhagic illness of children that begins with conjunctivitis and has a case-fatality rate of 40%–90% (*1**,**2*), was first recognized during a 1984 outbreak. Before June 2007, 69 cases were reported worldwide; 65 were from Brazil (*1**–**3*). To our knowledge, the disease had not been reported in the Amazon region until this investigation, which was precipitated by the report of 5 cases of a compatible syndrome in Anajás, Pará State, Brazil, in August 2007.

To determine whether recent reports of BPF were accurate, we reviewed medical records of the hospital in Anajás. We identified cases by using the following definition: fever >38.5ºC , abdominal pain, vomiting, purpura, and antecedent conjunctivitis during July 1–September 30, 2007, in a child 3 months–10 years of age; absence of signs or symptoms of meningitis in those children; and laboratory exclusion of meningococcal infection. In addition, we searched retrospectively and prospectively for conjunctivitis among pupils of the elementary schools of Anajás during July–September 2007. We found 7 children with illnesses that met our case definition.

From 2 children with nonfatal illness, we collected blood, serum, conjunctival swabs, and cerebrospinal fluid (CSF). All specimens were submitted for bacterial culture in half agar chocolate without bacitracin; serum and CSF were also subjected to real-time PCR for detection of *Neisseria meningitides,* *Streptococcus pneumoniae,* and *Haemophilus influenzae* serotypes a, b, c, and d and to conventional PCR for the *ompP2* gene of *H. influenzae*. All serum samples were also tested by hemagglutination inhibition for Flavivirus, Oropouche, Catu, Caraparu, Tacaiuma, Mayaro, Mucambo, western equine encephalitis, eastern equine encephalitis, Guaroa, Maguari, Ilhéus, Rocio, and St. Louis encephalitis; by immunoglobulin M antibody-capture ELISA for dengue and yellow fever; and, when reactive for dengue, by reverse transcription–PCR for dengue types 1, 2, 3, and 4.

Because of the remoteness of the outbreak site, samples for bacterial culture were collected on locally available blood agar enriched with rabbit serum without antimicrobial drug–selective agents, rather than on the recommended chocolate agar enriched with horse serum and bacitracin (*1*). Samples were transported over several days by open boat at ambient temperature (≈35 ºC) in improvised containers without an incubator. Serum and CSF samples were thawed and refrozen repeatedly for removal of aliquots before testing. Microbiologic and virologic testing was conducted at the Pará State Health Laboratory and Evandro Chagas Institute. Serum and CSF samples were tested by PCR at Adolfo Lutz Institute.

We identified 7 case-patients (median age 4 years, range 2–8 years): 6 from review of charts at the local hospital and 1 from active search in the rural community. Onset of illness was August 1 for the first case-patient and August 31 for the last. Five (71%) did not receive antimicrobial drugs and died within 24 hours after fever onset; 2 were treated with amoxicillin within 24 hours after fever onset and survived (Table).

**Table:** Characteristics of 7 case-patients with suspected Brazilian purpuric fever, Amazon region, Brazil, August 2007*

Case- patient no.	Sex/age, y	Date of conjunctivitis onset	Date admitted to hospital	Hospitalization, d	Antimicrobial drug treatment	Date of death	Type of sample(s) collected	WBC/mL
1	M/2	Aug 1	Aug 5	<1	No	Aug 5	NC	NT
2	F/3	Aug 10	Aug 13	<1	No	Aug 14	Blood	3,700
3	M/3	Aug 14	Unknown	Unknown	No	Aug 21	NC	NT
4	M/6	Jul 19	Aug 22	<1	No	Aug 23	Blood	2,300
5†	M/8	Unknown	Sep 3	7	Yes	–	CSF, conjunctival swab	9,060
6	F/4	Aug 23	Aug 26	<1	No	Aug 26	CSF, blood‡	2,000
7	F/4	Aug 31	Sept 3	12	Yes	–	Oropharyngeal and conjunctival swabs, CSF, blood, serum	4,800

*Case-patients 5 and 7 underwent testing for dengue and yellow fever by immunoglobulin M antibody-capture ELISA, for dengue by reverse transcription–PCR, and for *Neisseria meningitidis*, *Streptococcus pneumoniae,* and *Haemophilus influenzae* serotypes a, b, c, and d and conventional PCR for detecting the *ompP2* gene. All tests were negative. WBC, white blood cells; NC, not collected; NT, not tested; CSF, cerebrospinal fluid. †Sample collected 20 days after admission. ‡Non-standard collection, transport, storage, or processing.

Laboratory tests showed leukopenia on the day of hospital admission (Table). All case-patients had antecedent conjunctivitis. All except the first case-patient had had physical contact with a previous case-patient through school or family; 5 were related (siblings or cousins). The period from exposure to onset of fever was 8–21 days.

Of 1,598 elementary school pupils investigated for conjunctivitis, 111 (7%) reported symptoms of conjunctivitis during July–September 2007. Reported treatment included home remedies and a nonprescription, locally available, cream containing a sulfa drug. Review of municipal hospital records during June 1–September 30, 2007, identified no additional cases of conjunctivitis. After the last case-patient died, 17 other persons were identified with purulent conjunctivitis: 4 at the municipal hospital and 13 during active case-finding in schools and the community. All were treated with oral amoxicillin and chloramphenicol optic solution, and 76 contacts were treated prophylactically with oral rifampin. No further suspected BPF cases were detected. Test results for acute arbovirus infection and PCR were negative for all patients (Table).

This outbreak of highly fatal illness is clinically compatible with BPF. Compatible features included young age, antecedent purulent conjunctivitis, signs and symptoms (i.e., antecedent conjunctivitis, fever 39.5Cº–41.0Cº, abdominal pain, nausea, vomiting, petechiae or ecchymoses), and high case-fatality rate. Epstein-Barr infection has reportedly produced similar symptoms (*4*) but with an illness lasting >7 days in contrast to the <24 hours for our case-patients.

We did not detect *H. aegyptius* in peripheral blood by culture or in serum or CSF by PCR in the 2 surviving children and in contacts of case-patients. One reason could be the remoteness of the investigation site, which resulted in improper sample collection, storage, and processing in the field before samples reached reference laboratories. Hemagglutination tests for arboviruses have low specificity. Therefore, another known or novel pathogen could have caused these cases.

Timely treatment with antimicrobial drugs for patients with suspected disease, prophylaxis of contacts, and treatment of children with conjunctivitis resulted in no additional cases. Intensive surveillance for febrile illness preceded by conjunctivitis, immediate treatment, contact prophylaxis, and appropriate prompt laboratory testing are essential for continued control of BPF in this region.
